# Enhanced Visual Temporal Resolution in Autism Spectrum Disorders

**DOI:** 10.1371/journal.pone.0032774

**Published:** 2012-03-21

**Authors:** Christine M. Falter, Mark A. Elliott, Anthony J. Bailey

**Affiliations:** 1 Department of Clinical and Developmental Neuropsychology, University of Groningen, Groningen, The Netherlands; 2 Department of Psychiatry, University of Oxford, Oxford, United Kingdom; 3 Center for Applied Perceptual Research, Kyushu University, Fukuoka, Japan; 4 School of Psychology, National University of Ireland, Galway, Ireland; 5 UBC Institute of Mental Health, Department of Psychiatry, University of British Columbia, Vancouver, Canada; Duke University, United States of America

## Abstract

Cognitive functions that rely on accurate sequencing of events, such as action planning and execution, verbal and nonverbal communication, and social interaction rely on well-tuned coding of temporal event-structure. Visual temporal event-structure coding was tested in 17 high-functioning adolescents and adults with autism spectrum disorder (ASD) and mental- and chronological-age matched typically-developing (TD) individuals using a perceptual simultaneity paradigm. Visual simultaneity thresholds were lower in individuals with ASD compared to TD individuals, suggesting that autism may be characterised by increased parsing of temporal event-structure, with a decreased capability for integration over time. Lower perceptual simultaneity thresholds in ASD were also related to increased developmental communication difficulties. These results are linked to detail-focussed and local processing bias.

## Introduction

The core symptoms of autism spectrum disorders (ASD) lie in abnormal reciprocal social interaction, communication, and stereotyped behaviours [1]. Additional secondary dysfunctions, mainly in the sensory and perceptual domain, seem to be the norm rather than the exception [2]. Moreover, performance on some cognitive tasks is characterised by superior ability. For instance, it has been suggested that individuals with ASD show superior performance in figure-disembedding [3], [4]. Superior performance in perceptual tasks has been attributed to a cognitive bias towards local processing of stimulus details, at the expense of processing global structure. This feature of autistic performance is termed Weak Central Coherence, WCC [5]. Although research has yielded equivocal results with respect to inferior processing of global structure [6], [7], superior local processing has been robustly demonstrated [8].

A perceptual function only recently tested in ASD is interval timing. Several recent studies suggest interval timing abnormalities in ASD (e.g., [9], [10], [11], [12], [13]; for a review see [14], [15]), whereas others report no group differences in timing tasks [16], [17], [18]. A different timing function, temporal event-structure coding, i.e. the coding of events as simultaneous/asynchronous and the perception of their relative order, has been under-researched in ASD but might potentially be relevant, as outlined below.

It was originally Brecher [19] who reported that very short temporal periods, in vision on average 57 ms, represent what he termed *units of subjective time*. Events falling within this time window would be perceived as simultaneous even if they were physically asynchronous; whereas events falling within two different units of subjective time would be perceived as asynchronous. If these units of subjective time are altered (as for instance in individuals suffering from first episode psychosis, e.g., [20]), then the processing of temporal event-structure would be compromised, which could in turn adversely affect higher-order functions that rely on accurate sequencing of events, such as action planning and execution [21], [22], language processing [23], and social interaction [24], all functions that are impaired in ASD.

A few studies seem to suggest impaired event-structure coding in ASD. Although not explicitly a perceptual study, Bennetto, Pennington, and Rogers [25] reported impaired memory for temporal order in individuals with ASD. In contrast to their finding of abnormal temporal order memory, participants with ASD performed normally on a task that tested stimulus recognition rather than memory for stimulus order. More recently, it has been shown [26] that individuals with ASD were significantly impaired in naming familiar objects when they were moved behind narrow slits. The authors interpreted their results as evidence of a failure to integrate details over time into coherent wholes. In the current study, we have directly tested temporal event-structure coding in ASD and related performance to developmental communication and social interaction deficits, because of the relevance of timing for language processing [23] and social interaction [24].

We measured perceptual simultaneity thresholds, i.e. the largest time difference between two stimuli at which the stimuli are still experienced as simultaneous. Thresholds of perceptual simultaneity between simple paired visual events have typically been reported to lie between about 30 and 60 ms, depending on the exact task used to measure the thresholds [27], [28], [29], [30], [31]. In the current study, perceptual simultaneity thresholds were obtained using a similar psychophysical procedure to that previously used to assess temporal event-structure coding in healthy participants [27], patients with schizophrenia [32], [33], patients during first episode psychosis [20], and children with developmental dyslexia [34].

The perceptual simultaneity task used in these studies required participants to judge whether two target bars changed luminance simultaneously or asynchronously whilst stimulus onset asynchrony (SOA) was systematically varied. Psychometric functions describing the relationship between percentage of ‘simultaneous’ responses and SOA were obtained for each participant. The sensory threshold is given by the inflection point of the psychometric function, which in this task is the temporal gap between stimuli above which a participant is able to detect asynchronous stimulus onsets. The slope or steepness of the psychometric function around the threshold indicates the rate at which detection performance changes as a function of increasing SOA. Given previous findings suggesting a deficit of temporal event-structure coding in ASD [25], [26] we predicted abnormal perceptual simultaneity thresholds in ASD.

## Methods

### Participants

Twenty adolescents and adults with a clinical diagnosis of ASD (mean age = 24 years, SD = 7 years) were selected from amongst participants in previous ASD studies at the University of Oxford and through advertising. ASD diagnoses were confirmed using the Autism Diagnostic Interview – Revised, ADI-R [35] and the Autism Diagnostic Observation Schedule – Generic, ADOS-G [36]. Verbal, performance, and full scale IQ scores were obtained using the Wechsler Abbreviated Scale of Intelligence, WASI [37]. Individuals were not included if they had a diagnosis of a comorbid psychiatric disorder, were taking any medication, or scored below 70 on the WASI. The ADI criteria for age of onset was not met by two of the participants with Asperger syndrome and two others scored one point below threshold on one ADI algorithm domain. Nevertheless, these four participants scored above the Autism cut-off on the other ADI domains and above the ASD cut-off on the ADOS-G algorithm and were included in the final analysis. Three clinical participants were not included in the final analysis (one because of falling below ADI algorithm cut-offs in multiple domains and two because of IQ scores below 70). One participant did not complete the perceptual simultaneity task because of tiredness. Consequently data from 16 participants with a diagnosis of Asperger syndrome (N = 12) or high-functioning autism (N = 4) according to DSM-IV-TR [1], were analysed for the perceptual simultaneity task.

Seventeen TD participants (mean age = 26 years, SD = 7 years) were recruited either through poster advertisements or through their prior involvement in studies at the University of Oxford. Exclusion criteria for the TD group were psychiatric diagnoses, use of any medication, and an IQ score below 70. One TD participant performed at chance level in the perceptual simultaneity task and was excluded from further analysis. Consequently, data from sixteen TD control participants were analysed.

Participants from the ASD group and TD groups were matched by chronological age, verbal IQ, and performance IQ scores (Table 1). There were no significant differences between groups on these measures (all t<1). All participants had normal or corrected-to-normal vision. Written informed consent was obtained from all participants (or parents where applicable) prior to any testing and ethics approval for the study was obtained from the National Research Ethics Service (NRES) UK. A large subgroup of individuals participating in this study also participated on the same day in a study of duration perception and attentional control, and on different testing days in MEG studies of temporal and Gestalt processing (unpublished data).

**Table 1:** Demographic data.

	ASD(N = 16; 1 female;1 left-handed)			TD(N = 16; 2 females;2 left-handed)		
	Mean	SD	Range	Mean	SD	Range
**Age**	24∶2	7∶0	16∶9–38∶3	26∶2	7∶4	14∶10–38∶6
**VIQ**	111	15	70–127	108	11	95–139
**PIQ**	114	12	92–136	114	8	104–125
**FIQ**	114	13	89–131	112	9	100–133
**ADI-A**	18	5	10–28			
**ADI-B**	15	4	9–21			
**ADI-C**	6	3	2–12			
**ADOS-A**	3	1	1–7			
**ADOS-B**	6	3	1–12			
**ADOS-C**	1	1	0–4			

Means, standard deviations, and ranges of age (years:months), verbal IQ (VIQ), performance IQ (PIQ), and full IQ (FIQ) of participants with an autism spectrum disorder (ASD) and typically-developing (TD) participants. ADI-R Social Interaction Domain (ADI-A), ADI-R Communication Domain (ADI-B), ADI-R Repetitive Behaviours Domain (ADI-C), ADOS-G Communication Domain (ADOS-A), ADOS-G Reciprocal Social Interaction Domain (ADOS-B), and ADOS-G Stereotyped Behaviours and Restricted Interests Domain (ADOS-C) of participants with ASD.

### Apparatus

Participants viewed stimuli from a distance of 60 cm on a 16″ desktop PC in an environment of ambient light (90 lux), so as to reduce the impact of onscreen persistence. Stimuli were presented using Inquisit 3 Software [38] at a refresh rate of 120 Hz.

### Stimuli and Procedure

In each trial a white fixation cross was presented centrally and remained on screen throughout the trial. Participants were asked to look at the fixation cross and to avoid eye movements or blinks during stimulus presentations. After 500 ms two grey vertical bars subtending 4.1×0.4 degrees of visual angle (11.1 degrees between their centres) were presented on a black background (3.5 lux) to the left and right of the fixation cross (with counterbalanced presentation location). The two bars' luminance was increased incrementally (5.2, 12.8, 22.6, 34.2, 53.7 lux) within 5 frames (42 ms), either simultaneously or with one of 12 SOAs defined by the monitor refresh rate, resulting in SOAs across the range 1–12 * 8.33 ms. In a two-alternative forced-choice procedure, participants had to indicate whether the two bars appeared simultaneously or asynchronously by pressing one of two keys on the computer keyboard. The bars remained on screen until a response was given. No feedback on performance was given. Participants completed a practice block of 10 trials followed by five experimental blocks consisting of 52 trials each. Each level of SOA was tested on 20 occasions with presentation order pseudo-randomised on a session-wise basis.

## Results

The analysis was performed on percentages of ‘simultaneous’ responses to each of the SOA levels. As the percentage data were not normally distributed, non-parametric statistics are reported throughout. Medians and quartile deviations of ‘simultaneous’ responses for both groups are shown in Table 2. The percentage of ‘non-simultaneous’ responses in the SOA = 0 ms condition represents the general guess rate for each individual. A median guess-rate of 5% (±5%) in the TD group and 7.5% (±5%) in the ASD group was apparent on preliminary inspection of the data. Therefore, individual data was corrected using the following probability-based correction (after [27]):
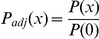
where *P (0)* represents the percentage of ‘simultaneous’ responses for an SOA of 0 ms (i.e. simultaneous).

**Table 2:** Percentage of ‘simultaneous’ responses across SOA.

SOA (ms)													
	0.00	8.33	16.66	25.00	33.33	41.66	50.00	58.33	66.66	75.00	83.33	91.66	100.00
**TD**	100.00	100.00	100.00	94.74	71.71	58.36	25.66	20.00	16.72	10.00	5.41	5.41	5.72
	(0.00)	(2.67)	(0.63)	(3.19)	(10.11)	(17.60)	(19.45)	(12.08)	(10.26)	(11.94)	(5.25)	(8.42)	(7.24)
**ASD**	100.00	100.00	97.50	81.67	55.00	40.59	6.11	5.72	6.27	2.50	0.00	5.13	0.00
	(0.00)	(3.33)	(3.33)	(7.09)	(18.71)	(17.61)	(18.03)	(8.75)	(11.76)	(6.97)	(4.29)	(3.97)	(0.66)

Bias-corrected means (SD) of ‘simultaneous’ responses (%) across stimulus onset asynchronies (SOA) of ASD and TD participants.

**Figure 1:**
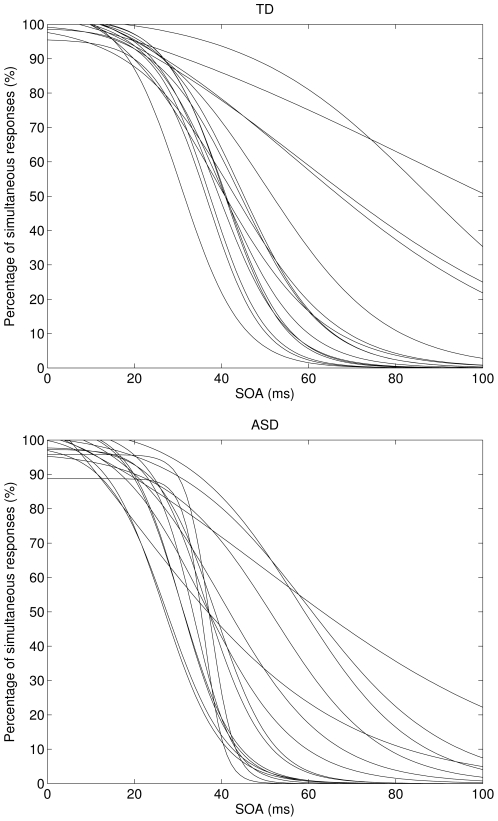
Individual curve fits. Sigmoid curves fit to the data of individuals from the TD group (top panel) and individuals with ASD (bottom panel) over stimulus onset asynchrony (SOA).

Subsequent to bias correction, a logistic curve was fitted (using the least squares procedure of [39]; adapted for Matlab by [40]) to individual data across SOAs in order to calculate individual slopes and points of inflection. The steepness of the slope represents the response criterion (the steeper the slope the sharper the distinction between the two response categories) and the point of inflection indicates the sensory threshold of simultaneity. The goodness of fit was high with *R^2^* = 0.94 (±0.06) in the TD group and *R^2^* = 0.96 (±0.04) in the ASD group. Figure 1 depicts fitted curves for all participants.

Simultaneity thresholds in the TD group (median = 41.92±6.53) were higher than simultaneity thresholds in the ASD group (median = 36.41±5.97). This difference was statistically significant in an undirected Mann-Whitney U-Test (*U* = 67.5, *p* = 0.022, *r* = −.40). The difference in thresholds was not due to a few outliers: the thresholds of eight ASD individuals were smaller than 0.84 SD below the mean of the TD group (i.e. their thresholds were below the 20^th^ percentile of the TD group) as compared to one person who showed such a low threshold in the TD group. This difference in distribution was significant in an undirected chi-square test (*χ^2^*(1) = 7.58, *p* = 0.006, *Yates' corrected p* = 0.015). In a further analysis we tested whether this effect was robust if the analysis was confined to individual thresholds with a goodness of fit of at least *R^2^* = .90. Three individual data sets from the TD group and one from the ASD group that did not meet this stricter goodness of fit criterion were excluded from this analysis. Applying the stricter criterion, the effect was robust and the threshold difference was still significant (*U* = 52.5, *p* = 0.038, *r* = −.39)

Slopes in the TD group (median = −2.67±0.95) were less steep than slopes in the ASD group (median = −3.67±1.14). This difference was not statistically significant (*U* = 92.0, *p* = 0.184, *r* = −.24).

In order to test whether thresholds were related to communication impairments, we calculated Spearman correlations between social interaction difficulties (ADI-A) and communication difficulties (ADI-B) with thresholds in the ASD group. While the ADOS-G only captures current communication skills, the ADI-R captures early development of communications skills as well as current skills.

**Figure 2:**
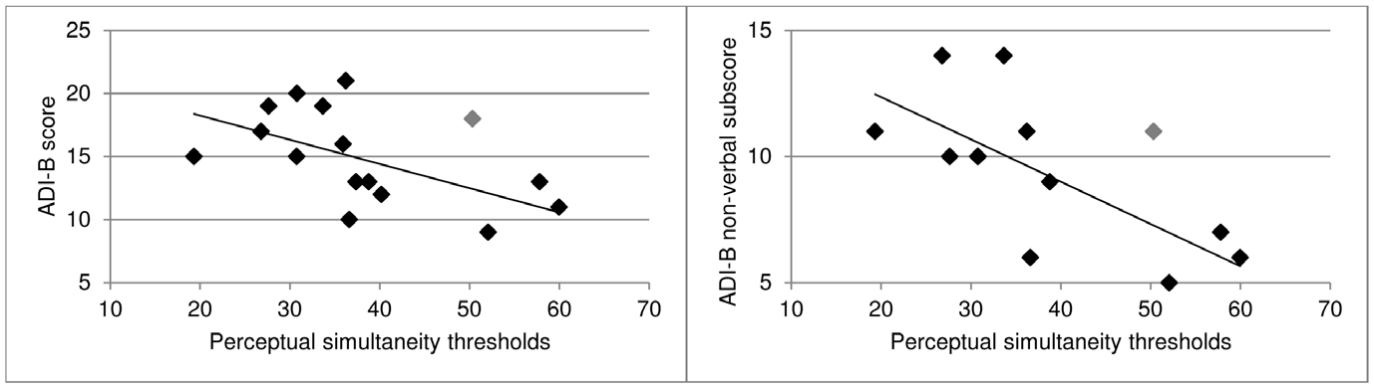
Correlation of thresholds with symptoms. ADI-B scores (left) and non-verbal ADI-B sub-score only (right) in the ASD group over perceptual simultaneity thresholds. The data point corresponding to the threshold derived from a curve fit with a goodness of fit *R^2^*>90 is depicted in grey. Note that there are two overlapping data points (31, 10).

The analysis showed that thresholds were significantly correlated with ADI-B scores (*r* = −.582, *p* = .018) only. Again, in order to test the robustness of this relationship, we repeated the analysis for those thresholds derived from curves with a goodness of fit of at least *R^2^* = .90. This stricter criterion even emphasised the effect more (*r* = −.679, *p* = .005). These results (see Figure 2) show that the more severely individuals with ASD are affected in the communications domain (i.e. higher ADI-B scores), the lower the perceptual simultaneity thresholds (i.e. the more deviant from the thresholds in the TD group). We tested what particular aspect of communication might be related to visual simultaneity thresholds in thirteen individuals for who individual sub-scores of the ADI-B were available. This additional analysis showed that it was the non-verbal aspects of communication (B1+B4 sub-scores) that were correlated with visual simultaneity thresholds (*r* = −.649, *p* = .016). These non-verbal sub-scores reflect communicative gestures (e.g., nodding, head shaking) and communicative functions based on social imitation (e.g., spontaneous imitation of actions).

## Discussion

Temporal event-structure coding was investigated in ASD using a perceptual simultaneity task. Individuals with ASD exhibited an enhanced resolution of temporal event-structure. The ASD group showed lower simultaneity thresholds compared to the TD group, even after correction for response bias. This study adds to previous reports of abnormal temporal event-structure coding in a range of patient populations (e.g., [32], [33], [20]). Importantly, however, the current findings show that individuals with ASD had significantly *decreased* perceptual simultaneity thresholds, rather than elevated thresholds as found in several other patient populations. Thus, increased temporal resolution might be a signature of the autistic cognitive profile, differentiating it from other developmental and psychiatric disorders.

As predicted, developmental communication impairments indexed by the ADI-R communications domain (ADI-B) were significantly related to thresholds in the ASD group; individuals with increased communication problems showed lower thresholds, whereas individuals with better communication skills and less impairment showed thresholds more similar to those in the TD group. This finding indicates that abnormal temporal event-structure coding is related to communication-related difficulties in ASD, similar to previous reports of impairments of individuals with developmental dyslexia in visual, auditory, and tactile simultaneity tasks (e.g., [41] but see [34]). Interestingly, lower simultaneity thresholds, i.e. better temporal resolution, did not serve as a protective factor for communication development in ASD. One could argue that deviations from the typical perceptual *units of subjective time* [19] or perceptual moments (von Baer, 1864 as cited and described in [30]) might lead to difficulties in communication. An adequate processing of temporal cues in the communication with another person might require an optimally tuned perceptual resolution rather than a perceptual resolution that is as low as physiologically possible.

Previous studies on temporal order judgements found significant impairments in ASD for auditory and cross-modal temporal order judgements, but no significant group differences in the visual domain [42], [43]. Although not significant, the visual temporal order thresholds in the ASD group were nominally lower than those in the TD group, which is in line with our findings. Nevertheless, these previous temporal order judgement studies [42], [43] are not readily comparable to the simultaneity judgment task in the current study. First, different sensory modalities exhibit different simultaneity thresholds [28], [29], [44], indicating different lower-level mechanisms involved in auditory versus visual temporal event-structure coding. Second, judgements of temporal order have been suggested by previous research to be distinct from judgements of simultaneity [45], [46] based on the finding that, in contrast to simultaneity judgements, thresholds of temporal order have been found to be often very similar across the auditory and visual modality [45]. Interestingly, and in line with these previous findings, Kwakye et al. [43] reports similar visual and auditory temporal order judgements in the TD group. In contrast, their ASD group showed profoundly different temporal order thresholds between modalities corroborating the authors' proposal of an alteration of temporal processing in ASD compared to TD. Indeed, such large differences in resolutions of temporal processing in the visual and auditory domain in the ASD group might well have led to their finding of extended temporal windows of integration for cross-modal processing [42], [43].

Our results are in keeping with Nakano et al's [26] suggestion that individuals with ASD are impaired in the integration of visual features over time. The current results expand upon those previous findings and show that perception in ASD is characterised by increased temporal resolution in vision, which can be interpreted as enhanced processing of detail in time. Thus, it seems that individuals with ASD are not only impaired in visual temporal integration [26] but also superior at temporal resolution. Together these findings are in line with WCC theory. They also provide an extension of the theory from the spatial into the temporal domain with at least one general implication for information processing that is consistent with WCC. In this case and as a consequence of finer temporal resolution, the ability to generalise beyond information in the attentional spotlight may be impaired simply because there is insufficient time available in the processing interval to recruit more neurons into the network than those currently representing visual information in attention. Clearly, further research into the relationship between the development of temporal event-structure coding and verbal development would be valuable. In addition, it will need to be investigated how temporal event-structure coding on a millisecond scale might translate into other higher-order functions which partially operate on larger timescales.

In conclusion, we found increased resolution of temporal event-structure in high-functioning individuals with ASD in terms of lower visual simultaneity thresholds. In this sense, individuals with ASD might speculatively show stronger parsing in time and weaker integration over time. As a consequence, individuals with ASD might experience less integration of past with present events with a potential detrimental impact on the content structure of conscious experience [47].
